# Living with a recalled implant: a qualitative study of patients’ experiences with ASR hip resurfacing arthroplasty

**DOI:** 10.1186/s13037-020-00278-y

**Published:** 2021-01-06

**Authors:** Christian Bitar, Ferid Krupic, Li Felländer-Tsai, Sead Crnalic, Per Wretenberg

**Affiliations:** 1 Department of Molecular Medicine and Surgery, Karolinska Institutet, Stockholm, Sweden; 2 Department of Orthopedics, Karolinska University Hospital, Stockholm, Sweden; 3 Department of Orthopaedics, Institute of Clinical Sciences, Sahlgrenska Academy, University of Gothenburg, Gothenburg, Sweden; 4 Division of Orthopedics and Biothechnology, Department of Clinical Science, Intervention and Technology, Karolinska Institutet, Stockholm, Sweden; 5 Department of Orthopedics, Karolinska University Hospital, Stockholm, Sweden; 6 Department of Surgical and Perioperative Sciences (Orthopaedics), Umeå University, Umeå, Sweden; 7 Department of Orthopaedics, School of Medical Sciences, Örebro University and Örebro University Hospital, Örebro, Sweden

**Keywords:** Articular surface replacement, Total hip replacement, Hip resurfacing arthroplasty, Medical device recall, Qualitative research, Interviews, Healthcare providers, Prosthesis failure

## Abstract

**Background:**

Total hip arthroplasty is the traditional treatment for osteoarthritis in the hip joint. Hip resurfacing arthroplasty, with metal on metal bearing, is a modern concept initially developed mainly for young active people. The metal-on-metal hip arthroplasty implant, Articular Surface Replacement (ASR), was implanted in approximately 93,000 patients before it was recalled in 2010 due to a high complication rate. This study aimed to evaluate patients’ own experiences living with an implant that they knew had a high complication rate and had been recalled from the market.

**Methods:**

A total of 14 patients, still living with the implant, of a cohort of 34 patients were available for follow-up. Qualitative semi-structured interviews were conducted with 14 patients where a majority actively sought for metal-on-metal hip resurfacing arthroplasty (HRA), and subsequently underwent HRA with an ASR prosthesis between 11/21/2006 and 09/28/2009. The responses were analyzed using content analysis described by Graneheim and Lundman to compress text and identify categories and subcategories.

**Results:**

The results showed that most patients had already decided that they wanted a metal-on-metal HRA implant before meeting the surgeon. They expressed that the implant made it possible to live an active life. A majority did not think about the fact that they had a hip implant, because they lacked subjective pain. Most of the patients were positive about the annual exams at the hospital and wanted them to continue. None of them felt that their trust towards the healthcare system had changed after the implant recall. They expressed a belief that they would need new surgery sooner than they first thought.

**Conclusions:**

Despite all the attention when the ASR prosthesis was recalled, patients with ASR-HRA did not report themselves negatively affected by the recall in this group of patients where a majority had actively sought for an HRA procedure. The healthcare system has an obligation to continue the annual exams, even if the implant provider does not continue reimbursement.

**Supplementary Information:**

The online version contains supplementary material available at 10.1186/s13037-020-00278-y.

## Background

Medical devices and implants are continuously developed and improved. However, patients receiving them are often unaware of the background and track record of the implants. When an implant has a high failure rate, it will sooner or later be recalled. After a recall, each patient will want to know how it will affect them medically [1].

The present study focuses on a recalled metal-on-metal hip arthroplasty implant, Articular Surface Replacement (ASR) by DePuy/Johnson&Johnson (Leeds, United Kingdom). The implant was developed in two options, hip resurfacing arthroplasty (HRA) and total hip arthroplasty (THA). From the introduction of the ASR hip system in July 2003 to its recall in August 2010, approximately 93,000 patients received this implant worldwide, including both HRA and THA [2]. Since 1996, more than 1,000,000 different metal-on-metal hip implants have been implanted worldwide [3].

After a few years with the ASR implant, there were alarming signs of complications from patients and joint registries. Among others, the UK joint registry showed high complication rates, and the implant was recalled in August 2010 [4–7].

The increased numbers of revisions of metal-on-metal HRAs and THAs are mainly due to 3 complications: mechanical (femoral neck fractures, implant loosening or luxation/subluxation), adverse local tissue reactions (ALTRs) (i.e., pseudotumors) and an elevated concentration of metal ions, such as cobalt (Co) and chromium (Cr), in the blood [8–11]. ASR failure has its origins, among others, in the acetabular component design (subhemispheric and shallow) with reduced acetabular cover, which increases the risk for edge wear [12–15].

In Sweden, 2143 patients received different metal-on-metal hip implants between 2000 and 2018 [16]. Birmingham hip resurfacing (BHR, Smith and Nephew, Warwick, United Kingdom), ASR and Durom (Zimmer, Winterthur, Switzerland) comprised 95.4% of all these implants [17]. A total of 396 persons received the ASR hip resurfacing arthroplasty and 169 persons received the ASR total hip arthroplasty. Most of the 396 patients in Sweden who received the ASR prosthesis are still living with it, with an 88% survival rate at 11 years [18]. Most patients received their prosthesis after a long struggle with osteoarthritis, only to learn later that the prosthesis was not as good as they thought it would be. In addition, they had to experience the adverse publicity connected to the withdrawal of the implant. These patients are regularly tested and checked via blood sampling and radiological examinations according to the national program for information and follow-up, which follows the international program recommendations [3, 11, 19].

Although the factors behind ASR failure are well -described, little is known about patients’ own experiences of having a recalled HRA implant. Therefore, it is interesting to explore the experiences of those who still live with their ASR prosthesis. The only qualitative study on a recalled implant in the literature was the study about the Poly Implant Prothése scandal, where a breast implant with a high complication rate was recalled. In the study [20], the author interviewed 12 patients and found that doctors and patients had different ideas on the gravity of a medical incident. This was evident in the difference between how doctors assessed the risk (low risk) of keeping the implant in relation to how the affected patients did. The author also found, that after a health scandal, the patients redefine their relationship with the healthcare system, which often led to distrust. Qualitative studies have been performed on patients’ experiences with THA, for example, experiences before and after THA [21], the experiences with preoperative information provided to patients [22] and experiences in fast-track hip and knee arthroplasty [23]. However, no qualitative study about patients’ experiences on a recalled hip implant has been performed earlier, and none on the ASR hip resurfacing arthroplasty implant.

The present study aimed to evaluate the patients’ experiences on receiving and living with an ASR prosthesis that they know has been recalled.

## Methods

### Study design

The study was designed as a qualitative study using data from interviews with patients who underwent HRA with the ASR prosthesis at Karolinska University Hospital in Solna, Sweden, between 11/21/2006 and 09/28/2009. The data were collected through individual semi-structured interviews with 14 patients.

### Patients

In total, 34 patients received the ASR implant at Karolinska University Hospital, 4 females and 30 males. A majority (9 patients) of the 14 interviewed patients had actively sought for HRA replacement procedure. Three of them had the implant bilaterally. As of June 2015, 3 patients underwent revision surgery.

We studied patients who underwent unilateral HRA with ASR prosthesis and had no need for revision surgery. Thus, 3 patients who underwent revision surgery prior to the study, and 3 patients who had bilateral implants were excluded from the study.

A research nurse at the Karolinska University Hospital, was instructed to choose patients from a list of 28 patients who underwent surgery at Karolinska University Hospital, according to the criteria above. All patients who were contacted agreed to participate.

The patients were 11 men and 3 women aged 44–83 (mean 62.1) years. The men were aged 44–83 (mean 61.5) years, and the women were aged 60–72 (mean 64.7) years. The patients lived in Stockholm County, and they came from 10 different municipalities of a total of 26 in the county. The patients had different socioeconomic status and geographical domicile. The demographic and clinical characteristics of the patients are shown in Table 1.

**Table 1:** Characteristics of the study population (*n* = 14)

Variables	No. of patients
**Gender**	
Male	11
Female	3
**Educational level**	
Elementary school	2
High school	5
University	7
**Mean age (years)**	44–83 (62.1)
**Age (years)**	
40–49	2
50–59	3
60–69	6
70–79	2
80–89	1
**Occupation**	
Employed	11
Unemployed	0
Retired	3

### Procedure

Data were collected through individual interviews by the first author (CB) using open-ended questions following an interview guide described by Kvale and Torhell [24]. All authors were involved in developing the interview guide. The interviews were conducted from February 2015 to June 2015 at the Karolinska University Hospital in Solna and Huddinge, and in one case, the interview was held at the patient’s home. The patients were asked to visit the hospital solely to participate in the interview and not as a part of a clinical appointment. There were no economical compensations for participation.

The first author of the study conducted the interviews and was trained in qualitative interviewing by the second author. The second author has much experience and expertise in qualitative research. The first author is an orthopaedic surgeon but had no part in the patient’s treatment or clinical care, thus minimizing bias and precluding a negative effect on how the patients responded during the interviews.

The first author initiated the interview by presenting himself professionally and informing the patients about the ASR research project. The patients were given information about the interview process, study layout and protection of confidentiality.

The patients were asked to speak freely and as comprehensively as possible. A general request opened the interview: “Tell me about yourself”.

They were asked questions about the key topics for this study, such as the following:

“Can you please tell me about your hip implant? What do you know about your implant? How does it feel to live with a high-risk implant? Have you been offered exams at the hospital? What are your thoughts about the future?” (Additional file 1).

The patients were asked to reflect on their experiences. The interviewer only interrupted for questions or to follow-up on information given. The patients were, for example, asked to clarify their thoughts. In some cases, they were asked probing questions to bring them back on topic. The interviewer often repeated their answer as a way of clarification and to give them more space for more exhaustive information.

The plan for this study was primarily to interview 10–20 patients. When no new information emerged during the interviews (after 12 patients were interviewed), saturation was met. We decided to stop including more patients, and we ended the interviews after the two that had already been planned.

All interviews were carried out in Swedish. The duration of the interviews was between 35 and 70 min, and the interviews were taped on both a digital and an analogue tape recorder. The data were transcribed verbatim.

### Data analysis

The material was analyzed using qualitative content analysis (QCA) according to Graneheim and Lundman [25]. QCA provides tools to analyze large amounts of data; find differences and similarities in the data; and compress text and identify units, themes, codes, categories and subcategories. With QCA, we were able to interpret the text, and the method gave us good knowledge of the manifest content and, to some degree, of the latent content [26].

The interviews were recorded and transcribed verbatim by a writing bureau. All the transcriptions were read by all study authors to get a complete picture of the patients’ experiences. In the next step, the texts were read separately, and meaningful units were found that were composed of related text that addressed a specific topic. The meaning units were condensed with a description close to the original text. The condensed meaningful units were labelled with a code. The codes were thereafter analyzed for differences and similarities, and similar codes were grouped together, which created subcategories. Finally, the subcategories were grouped together next to similar topics, whereby a conclusion about main categories was reached.

All authors were involved in the data analysis and coding of the data.

The results of the analysis were used, compared and completed by the first author (CB) of the study.

### Ethics approval

The study was approved by the Regional Committee for Ethics at Karolinska Institutet – Dnr: 2014/1067–31/3. After receiving oral and written information about the study, all patients signed a written consent form.

## Results

The analysis of the data resulted in three main categories and nine subcategories, according to how the patients experienced their situation of having an implant with a high complication rate. The categories were: receiving a new hip joint, recall of the ASR prosthesis and having a recalled prosthesis. The categories and subcategories are presented in Table 2 and are clarified by quotations from the interview texts.

**Table 2:** Categories and subcategories derived from the data analysis

Category	Subcategory
**Receiving a new hip joint**	Meeting the surgeon
	The prosthesis affected life
**Recall of the ASR prosthesis**	Information about the recall
	Experience of the recall
	Knowledge of the prosthesis
**Having a recalled prosthesis**	Annual exams
	Still living with the prosthesis
	Affected trust in healthcare
	Expectations/concerns

### Receiving a new hip joint

In the first main category, most of the patients described that they sought out a center that conducted hip resurfacing, due to the fact they did not want to be physically inhibited by a hip implant. The patients even described how they got their information about the implant and how much information about the implant choice they got from the surgeon. They even painted a picture of happiness with their implant and the fact that their hip pain was gone, even though some of them had some residual pain. The majority of the informants in the present study emphasized that they could return to an active lifestyle and even continue with their sports activities.

#### Meeting the surgeon

Almost all patients in the present study commonly described that they had largely decided that they wanted an HRA implant, even before they met the surgeon. Patients described that, even before the meeting with the surgeon, they did research regarding which hospitals conducted this type of surgery, they had been reading on the internet about the implant and they discussed it with others who had the same surgery.

“*I got good information, and then looked for it on the internet as well”.*

*“I read about the implant, I knew the method”.*

*“It was what I wanted, it was my choice”.*

The fact that the patients wanted to continue being active played a great role during the meeting with the surgeon. Accordingly, some patients were affected when the surgeon told them they were young and would benefit more from this kind of implant, and they would not be able to continue their sports activities with a traditional THR.

*“ … the risk that it would dislocate was smaller and because I wanted to continue to be able to ski, swim, bike and walk on the islands and all the other stuff, I began searching for who could do it”.*

According to the patients’ experiences, the information regarding the implant from the surgeons varied. Some of the informants had received information about both options, the traditional THA and HRA alternative, from the surgeon. Some of the remaining patients only received information about HRA. The choice of implant was, according to some of patients, the surgeon’s decision, and in a few cases, it was a mutual decision.

*“He said that the most common prosthesis is the traditional. But more recently, we have begun to use this new one to a greater extent, and it has worked well. From everything I had read online, many people were positive about it, so there was no more doubt”.*

#### The prosthesis affected life

A majority of the patients expressed their happiness that their hip pain was gone after surgery. They felt that the implant gave them an opportunity to live an active life. In all happiness, they described that their activities included yoga, working out at the gym, swimming, playing ice hockey, skiing, ice skating, cross country skiing, playing tennis, playing squash, running, sailing, walking, biking and gardening.

*“I am very physically active. I have also been able to get back to tennis. I play tennis, I jog, I ski and I bike. Half a year after the surgery, I actually completed Vasaloppet”.*

However, some of them were not able to run for exercise anymore, and some did not dare to do activities involving a risk of falling. Some of the patients described that they had some problems with their new hip. Pain in some angles, the implant making sounds in some angles, cramping in the leg occasionally and pain in the hip in some angles, were some of the symptoms and difficulties the patients observed after the surgery.

*“I can feel it sometimes as a kind of cramp now and then, especially when it is cold, but otherwise it is no worries at all. It is impossible to compare with when I had the bad hip, when I had incredible pain and was very limited. So, it’s like night and day”.*

### Recall of the ASR prosthesis

In this second main category, the patients stated that they got their information about the recall mainly from two sources, by letter and from their healthcare provider. At first, the patients were worried about the recall, but with time, most of them were reassured by the information they received and by their own research. The interview indicated that patients’ knowledge about why the implant was recalled was poor and focused mainly on a few complications. The greatest knowledge was about the elevated concentration of metal ions in the blood.

#### Information about the recall

The patients reported that they learned about the recall for the first time from a variety of sources. One of the most common ways was by a letter from Crawford & Company, which was an insurance and claims settler who was retained by Johnson & Johnson to handle the hip recall lawsuits and related injury claims. The other most common way was in a letter with an appointment for the surgeon and from the healthcare provider. The informants also expressed that, in some cases, it was through a phone call from a nurse at the hospital, and in other cases, it was directly from the surgeon. One patient heard about it from the media.

Some of the patients felt that it would have been better to get information from the hospital first, while others thought that it did not matter from where they first received the information. Those who preferred that the hospital should have provided the first contact meant that they would have received reassuring information at the same time and would not had to worry until they got all the information.

“ … they should have summoned all patients who had undergone surgery with this method, and inform them about it, for example what was the impact of it all and how to decide whether one should do revision surgery or not”.

“Yes, it would have been better to get it from the orthopaedic surgeon first of course, but I'm not so sensitive in that way”.

#### Experience of the recall

Most patients initially felt worried when they first got the news of the recall. They were worried that they would have to undergo another operation, they would not get to keep their implant and they would become ill because of the implant.

Later, when they received more information from the hospital, they were reassured. Among other things, they were reassured by the fact that, if they needed to undergo revision surgery, it would be easier to change the implant to a traditional one compared with having a THR from the beginning. However, some patients were shocked about the risk of pseudotumors and fractures. One male patient felt reassured by the information that mainly women were affected.

“But as I said, if it goes wrong, we have a second chance, in the way that we can remove this one, not us but someone can do it, and put in a traditional one”.

“ … as long as it feels good, I think it is okay. When it does not feel good anymore, then maybe one should worry”.

The majority of the patients concluded that the recall did not concern them since they had no symptoms from their hip.

Three patients never felt worried and did not care at all about the recall. In a few cases, the patients were sad about it, because they were happy with their implant. Patients claimed wanting a similar implant if the other hip caused problems in the future.

“If I need to do a revision surgery and if there is a similar prosthesis with the same principle, then I would like to have one of those”.

“Yes, but I felt really privileged, as it is clear that one feels cheated afterwards when you learn that the method was not good”.

#### Knowledge of the prosthesis

The patients in the present study had incomplete knowledge of the complications with the ASR prosthesis and which reasons led to the recall. Although most of the patients had some information about the implant, the experiences of the informants were that they had a low level of knowledge about the implant’s complications.

“I knew nothing about it, and I still don’t know what's wrong today”.

Barely half of the patients talked about the risk of elevated metal ions in the blood, even though they are tested annually via blood samples for Co and Cr. Some of the patients knew about the risk of implant loosening and the risk of fracture. However, many of these patients said that they did not know anything about the risks and reasons for the recall of the product.

“It releases metal in too high an extent and it has become loose in too large a number in order for one to be satisfied with it”.

### Having a recalled prosthesis

In this third main category, the patients explained that they were happy with the annual exams. It gave them comfort, and they hoped that the annual checks would continue in the future. The patients were not bothered by the fact that they had an implant with high complication risks, but some of them said they were a slightly more cautious so as not to trip and break their hip. Many of the patients hoped they would be able to live with the implant for a long time without any complications or pain. They were satisfied with their healthcare, and they felt cared for, seen and not forgotten by the hospital.

#### Annual exams

Most interview patients were happy with their annual exams, at least with the blood samples for testing Co and Cr concentrations. They felt that it gave them a sense of security. Initially, when the testing began, they were worried until they got the results back from the laboratory. However, after a couple of times, they felt cared for by the annual visits to the hospital. Many of them expressed that they were very happy with the nurse who handled the contact and administration during the exams and that it meant a lot.

“I was sad and worried, but then I was told that one could come for an appointment annually that they would check it all and it felt good”.

All patients in the present study wanted the annual exams to continue. They explained their views in different ways, mainly their opinion was that the healthcare system implanted it, so they should take care of the consequences. An interesting thought expressed from one of the patients was “the longer you have the prosthesis, the more worn out it would be, and therefore, the exams should continue”.

“I would probably have wanted the testing to be at least 10 years after surgery, but now maybe it will be eight … I do not know if there is a greater risk that there will be problems with this the older it gets, and one does not know much about that as it is not so old technology”.

“Yes, I would hope that KS (Karolinska University Hospital, Solna) continues this sampling or, I will in any case, require that they do so”.

#### Still living with the prosthesis

The majority of the patients in this study were currently not bothered by the recall of the implant. According to the patients’ experiences, the fact that they do not have any pain or problems with the implant makes them not concerned about the high complication rate of the implant. It seemed that they were happy with the past years where they could keep the implant, which made it possible to live an active life. Some of them were afraid that something would happen with the hip, such as if they were to fall. Others stated that they would replace the implant only if new evidence indicated that it was very dangerous to have the ASR implanted.

“Yes, I thought it worked well, and I could keep up much of my old activities. Then, when I was told that I would receive support from here, and if it looked bad, it would be operated on immediately, I felt right confident with it”.

” … during these years now, it has functioned so well, that I have received value from it”.

Their opinion was mirrored by what one of the patients who said, “Why should I think about it when it works for me?” Some of the patients also said that they think about their hip only when it beeps at the security check at an airport.

#### Affected trust in healthcare

All patients stated that their confidence in the healthcare system was not affected by the fact that they got a prosthesis that was later recalled due to a high complication rate. They were satisfied with the support they got from the hospital, which was open and straightforward with them. They also felt that they all got information that was available after the recall.

“ … from the healthcare and the orthopaedic surgeon, both the outpatient clinic and the ward, they have been very open and straightforward”.

#### Expectations/concerns

Most patients thought that they probably would need to have the implant changed in a shorter timeframe than was first intended. However, many of them still hope that they are the lucky ones and that the implant will function well for the rest of their lifetime. Some of them hoped that it would last at least another 10–15 years.

“There is a good risk that one will need to do something during a lifetime … that day, that grief”.

Some patients understood that they would get a THR when they needed to change the implant, but others still hope there is a similar implant with better survival that they can have implanted instead. Most of the patients state that they do not think about the future of the implant and are not worried.

“It is now in the sixth year, if I would have to do the right hip, then I definitely want to have a similar implant. Even if it now would only last, say, five years I would like to have it anyway, because I'm not as limited”.

## Discussion

The present study aimed to obtain a greater understanding of the patients’ own experiences of living with a recalled ASR implant. We wanted to understand, interpret, describe, and explain these patients’ experiences; therefore, the use of a qualitative descriptive approach was appropriate. For the analysis, QCA was an appropriate choice. With QCA, one is able to seek understanding and study the wholeness and be able to categorize and classify large amounts of text [26].

We found that patients were not negatively affected by the knowledge of having an implant with a high complication rate, as long as they did not have any problems. We also found that none of the patients felt that their trust towards the healthcare system had decreased after the withdrawal of the implant. We believe that the national program for information and follow-up for patients with ASR might be a reason for this outcome, and this finding highlights the importance of information. We believe that the results from this study could be useful for similar research and healthcare professionals, as we found that, if patients are taken care of in a professional manner after failures, their trust towards healthcare remains.

To our knowledge, there was no qualitative study previously done in this area, and we feel it is very important to obtain insight into the patients’ experiences with a recalled implant. In opposite to the study about the Poly Implant Prothése scandal by Greco [20], our study shows no distrust in the healthcare system. We could also not find any mismatch between the patients and doctors with regard to assessing the risk of keeping the implant in, contrary to what Greco et al. showed in their study.

Many of our patients read on the internet about, in their opinion, the positive attributes of metal-on-metal bearings, such as that they could continue being physically active. This benefit was, and to some extent still is, the general opinion and one of the motives for using metal-on-metal bearings [27–29]. This was one of the reasons our patients wanted this particular implant. However, only half of the patients received information regarding other THA options and not only about HRA. Although patients sought out the clinic specifically for the reason they wanted an HRA, the patients should have been given information about other options. According to other studies, patients express concern about inadequate preoperative information and point to the importance of it [22, 23]. Bautista et al. [30] indicated that the chosen implant for surgery should be followed up for at least 10 years or should have a minimum of 90% survival. These criteria were not met before the introduction of the ASR prosthesis, and the patients should have been informed of that fact.

The patients in the present study received the first information about the implant recall from different sources. Some and some of the patients were dissatisfied with the fact they did not get it from the hospital first, while others did not care who they heard it from first. Most patients were worried when they got the news but felt reassured when they met the surgeon and got information about the national follow-up program. Hence, if a company is affected by an implant recall, they should collaborate with the healthcare provider and give them a timeframe for informing patients before sending information to the patients [31]. Interestingly, in the UK, some of the patients did not get the recall notice at all [28].

Annual exams are very important for the patients. The follow-up gives patients a sense of security and shows that the healthcare system takes responsibility when something goes wrong. Indeed, the fact that the hospital was open and straightforward regarding the recall may be one of the reasons the healthcare system did not lose the patients’ trust in the present study. This finding is in line with findings from Banarjee and Mont [31], who noted that it is the implant company’s duty to inform the surgeons, but it is the surgeon’s duty to inform the patients and to arrange follow-up.

In Sweden, the implant company would reimburse the costs for metal ion testing until August 2017 and for revision surgery for up to 10 years after implantation. Thereafter, further blood sampling and annual exams would be conducted according to the decision made by each hospital. Our study showed that most patients were positive about the annual exams and wanted them to continue. We recommend that patients should be part of the decision process regarding continuation of the tests.

None of the patients in our study population had full knowledge of the possible complications of their implant. Knowledge was gathered about elevated ion levels, implant loosening and fractures. Abu Al-Rub et al. [32] showed that the knowledge patients have about their implants is low. They also showed that because patients do not know which implant they have, they are affected by the information in the media about metal-on-metal implants, even though they have a standard THA. We agree with Abu Al-Rub et al., who suggested that patients should get a card with information about their implant [32].

### Study limitations

One limitation is that the study was conducted only with patients who underwent HRA with ASR in Sweden, and therefore, it might be difficult to extrapolate the findings to a broader context. Since the majority of the patients had actively sought for an HRA procedure the results may not be generalizable to a general patient population. Another limitation is that the patients who received a THA (ASR XL) implant were not included in the study. Furthermore, the interviews took place several years after the recall, and patients may have forgotten the information they initially received [33].

## Conclusions

The results of this qualitative study show that despite all the attention when the ASR prosthesis was recalled, patients who did not need revision surgery did not seem to be adversely affected by the incident in this group of patients where the majority had actively sought for an HRA procedure.

The patients felt cared for with annual exams. For most patients, the exams were important and gave them a sense of security; the patients wanted them to continue. Healthcare providers should therefore give patients the option to continue with the annual exams.

## Supplementary Information

**Additional file 1.**

## Data Availability

No additional data are available due to the importance of confidentiality.

## Abbreviations

ASR: Articular Surface Replacement
HRA: Hip Resurfacing Arthroplasty
THA: Total Hip Arthroplasty
Co: Cobalt
Cr: Chromium
QCA: Qualitative Content Analysis
MoM: Metal-on-Metal
