# Supplementary material for: Living with a recalled implant: a qualitative study of patients’ experiences with ASR hip resurfacing arthroplasty
Source: Patient Saf Surg. 2021 Jan 6;15:2. doi: 10.1186/s13037-020-00278-y (PMC7788783; doi:10.1186/s13037-020-00278-y)
Supplement: Supplementary file 1 — Additional file 1. [file 13037_2020_278_MOESM1_ESM.pdf]

Interview questionnaire

- Can you please tell me about yourself?
- Do you have any hobbies?
- Do you have a life partner?

- Can you please tell me about your period of illness regarding to your hip?
- Can you please tell me about your hip implant?
- What can you tell me about the choice of implant?
- What information did you receive about the implant?

- You know that you have an ASR implant, that later was recalled. What do you know about the reasons for the recall?
- How did the recall affect you?
- How did this affect your relationship with healthcare?
- What do you know about your implant?
- How does it feel to live with a high-risk implant?

- Have you been offered exams at the hospital?
- How do you feel about the annual exams?

- Has all this with the ASR implant affected you economically in any way?
- What are your thoughts about the future?
- How does your daily life look like nowadays?
